# Isoform-specific AMPK association with TBC1D1 is reduced by a mutation associated with severe obesity

**DOI:** 10.1042/BCJ20180475

**Published:** 2018-09-25

**Authors:** Elaine C. Thomas, Sharon C. Hook, Alexander Gray, Alexandra Chadt, David Carling, Hadi Al-Hasani, Kate J. Heesom, D. Grahame Hardie, Jeremy M. Tavaré

**Affiliations:** 1 School of Biochemistry, Biomedical Sciences Building, University of Bristol, Bristol, U.K.; 2 Division of Cell Signalling & Immunology, School of Life Sciences, University of Dundee, Dundee, U.K.; 3 German Diabetes Center, Leibniz Center for Diabetes Research, Heinrich-Heine-University, Medical Faculty, Düsseldorf, Germany; 4 German Center for Diabetes Research (DZD), Düsseldorf, Germany; 5 Cellular Stress Group, Medical Research Council London Institute of Medical Sciences, Hammersmith Hospital, Imperial College, London, U.K.

**Keywords:** AMPK, glucose transport, obesity, protein–protein interactions, Rab-GAP, signalling

## Abstract

AMP-activated protein kinase (AMPK) is a key regulator of cellular and systemic energy homeostasis which achieves this through the phosphorylation of a myriad of downstream targets. One target is TBC1D1 a Rab-GTPase-activating protein that regulates glucose uptake in muscle cells by integrating insulin signalling with that promoted by muscle contraction. Ser^237^ in TBC1D1 is a target for phosphorylation by AMPK, an event which may be important in regulating glucose uptake. Here, we show AMPK heterotrimers containing the α1, but not the α2, isoform of the catalytic subunit form an unusual and stable association with TBC1D1, but not its paralogue AS160. The interaction between the two proteins is direct, involves a dual interaction mechanism employing both phosphotyrosine-binding (PTB) domains of TBC1D1 and is increased by two different pharmacological activators of AMPK (AICAR and A769962). The interaction enhances the efficiency by which AMPK phosphorylates TBC1D1 on its key regulatory site, Ser^237^. Furthermore, the interaction is reduced by a naturally occurring R125W mutation in the PTB1 domain of TBC1D1, previously found to be associated with severe familial obesity in females, with a concomitant reduction in Ser^237^ phosphorylation. Our observations provide evidence for a functional difference between AMPK α-subunits and extend the repertoire of protein kinases that interact with substrates via stabilisation mechanisms that modify the efficacy of substrate phosphorylation.

## Introduction

AMP-activated protein kinase (AMPK) is a key regulator of cellular and systemic energy homeostasis. Activation of the enzyme can be brought about by energy stress that is signalled by increases in intracellular AMP:ATP or ADP:ATP ratios, or by increases in intracellular Ca^2+^ ion concentrations [1,2].

Mammalian AMPK occurs as heterotrimeric complexes comprising α, β and γ subunits, each with distinct functions. Catalytic activity is conferred by two distinct genes, *PRKAA1* and *PRKAA2* which encode the α1 and α2 subunits, respectively [3]. These can form up to 12 different combinations of αβγ heterotrimer with the β1 and β2 subunits, and the γ1, γ2 and γ3 subunits. Phosphorylation of threonine 172, in both α1 and α2, is required for maximal activation of AMPK [4]. T172 phosphorylation can be induced by pharmacological activators of AMPK such as 5-aminoimidazole-4-carboxamide riboside (AICAR), and this is associated with increased glucose uptake [5–9] and translocation of GLUT4 glucose transporters to the plasma membrane [10] in skeletal muscle.

AMPK-mediated glucose uptake in skeletal muscle is thought to involve TBC1D1 [11,12], a Rab-GTPase-Activating Protein (Rab-GAP) closely related to AS160 (TBC1D4) [13]. TBC1D1 is highly expressed in glycolytic muscles [14–16], although recent evidence suggests that TBC1D1 may also play a role in additional tissues including the pancreas, liver and kidney [17–19]. Both TBC1D1 and AS160 possess two phosphotyrosine-binding (PTB) domains, a calmodulin-binding domain and a Rab-GAP domain [20]. While the functions of the TBC1D1 PTB domains are poorly understood, evidence from AS160 [21,22] and TBC1D1 [23] studies suggests regulatory and signalling roles. TBC1D1 is phosphorylated in response to AMPK activation on multiple sites, with the best characterised being Ser^237^, within the second PTB domain (PTB2), a covalent modification that promotes 14-3-3 protein binding [15,19,24,25].

Sequence variation in *TBC1D1* is associated with growth- and obesity-related traits in pigs, chickens and rabbits [26–29] as well as humans [30,31]. A coding polymorphism (rs35859249; R125W) found in the first PTB domain (PTB1) has been reported in two independent studies to be associated with rare forms of severe familial obesity in women [20,32]. Our homology model of this domain indicated that R125 is located in a putative peptide-binding cleft, suggesting a tryptophan substitution would alter protein–protein interactions [33]. However, how this single-point mutation manifests as a severe obesity phenotype is currently unclear.

The initial aim of the present study was to identify proteins that bind to the PTB domains of TBC1D1 using Stable Isotope Labelling by Amino acids in Cell culture (SILAC). We found a stable association of AMPK heterotrimers containing α1 but not α2 subunits and demonstrate that this has functional consequences for the kinetics of phosphorylation of the critical AMPK-directed phosphorylation site on TBC1D1, Ser^237^. We also demonstrate that this interaction is disrupted by the naturally occurring R125W mutation.

## Materials and methods

### Antibodies and reagents

Unless otherwise stated all reagents were from Sigma–Aldrich. Primary antibodies against pan-AMPK-α (#2532), AMPK-β1/2 (#4150), pAMPK Thr^172^ (#2531), AKT (#4685), ACC (#3876), pACC Ser^79^ (#3661), IRAP (#3808), rabbit FLAG (#2368), Raptor (#2280), pRaptor Ser^792^ (#2083) and TBC1D1 (#5929) were from Cell Signaling Technology. Other antibodies were pan-14-3-3 (sc-629) and GST (sc-138) from Santa Cruz Biotechnology Inc., GFP (118144600001; Roche), AMPK-γ1 (ab32508; Abcam), pTBC1D1 Ser^237^ (#07-2268; Merck-Millipore), β-actin (A1978) and mouse FLAG (F1804). Isoform-specific AMPK-α antibodies were described previously [34]. A769662 (Abcam) and AICAR (Tocris) were used.

### Mouse model

Transgenic MCK-3xFLAG-*Tbc1d1* mice with muscle-specific overexpression of *Tbc1d1* were generated by cloning of PCR-amplified 3xFLAG-*Tbc1d1* from skeletal muscle cDNA into pBSK-MCK containing exon, one of the muscle creatine kinase promoters. The construct was injected into the fertilised oocytes of FVB/N inbred mice (Prof. Fatima Bosch and Dr. Anna Pujol, UAT-CBATEG, Barcelona, Spain). A founder line with high (5-fold) *Tbc1d1* overexpression in heart and skeletal muscle tissue was backcrossed to the N7 generation with C57BL/6J mice using marker-assisted genotyping.

Mice were kept in accordance with the National Institutes of Health guidelines for the care and use of laboratory animals, and all experiments were approved by the Ethics Committee of the State Ministry of Agriculture, Nutrition and Forestry (State of North Rhine-Westphalia, Germany). Three to six mice per cage (Macrolon type III) were housed at a temperature of 22°C and a 12-h light–dark cycle (lights on at 6 a.m.) with *ad libitum* access to food and water. After weaning, animals received a standard chow diet (V153 3 R/M-H; Ssniff, Soest, Germany). Male mice between 10 and 14 weeks were killed by cervical dislocation, quadricep muscle was dissected and directly snap-frozen in liquid nitrogen.

### Molecular biology

TBC1D1 constructs and AS160 were cloned from pCMV5.HA-1 TBC1D1 and p3xFLAG-CMV-10 AS160 (kind gifts respectively from Kei Sakamoto, Nestlé Institute of Health Sciences S.A., Switzerland [25] and Gus Lienhard, Dartmouth Medical School, US) respectively, into pXLG3 for lentiviral expression or pEGFP-C1 for transient expression to yield constructs with an N-terminal GFP tag. Secondary structure prediction, described previously [33], determined PTB domain regions as residues 1-161 (PTB1), 162–381 (PTB2) and 1–381 (PTB1 + 2). QuikChange site-directed mutagenesis (Agilent Technologies) introduced point mutations, TCC to GCC (S237A), ATG to CTG (M232L), ATG to GCG (M232A), CGC to GCC (R233A), CTG to GCG (L241A), CGG to TGG (R125W) into TBC1D1 and AAG to AGG (L47R — kinase-dead mutation) into AMPKα1. The PTB1 + 2 domain of TBC1D1, wild-type and R125W mutant, was cloned into pGEX-6P-3 with a C-terminal Hexa-His-tag and a C-terminal Hexa-His-tag was cloned onto GST in pGEX-6P-3.

### Cell culture and generation of stable cell lines

C2C12 (American Type Culture Collection), Flp-In HEK293 (Invitrogen) and HEK293T cell lines were cultured in DMEM supplemented with 10% (v/v) FBS (Invitrogen), 50 IU/ml penicillin, 50 µg/ml streptomycin and 2 mM glutamine. C2C12 cells were differentiated into myotubes as previously described [35]. HEK293T cells were used to produce lentiviral particles which were added to C2C12 myoblasts to generate stably transduced cell lines. Flp-In HEK293 cell lines stably expressing either FLAG-tagged AMPK-α1 or -α2 were previously described [36].

### Generation of AMPK-α1/-α2 double knockout (DKO) HEK293 Flp-In cells

Knockout of AMPK-α1 and -α2 (*PRKAA1* and *PRKAA2*) in HEK293 Flp-In cells was performed via the CRISPR-Cas9 method [37] using the targeting oligos, methodology and validation as described previously [38]. As some residual activity was initially detected by enzyme assay at very low levels, probably due to the polyploid nature of the HEK cell line, the process was repeated on these cell lines to remove all detectable AMPK activity.

After transfection with FuGENE 6 (Promega), α1/α2 DKO cells expressing either FLAG-AMPK-α1 or FLAG-AMPK-α1 K47R were selected and single foci expanded in complete medium supplemented with blasticidin (15 µg/ml) and hygromycin B (200 µg/ml).

### SILAC interactome analysis

Transduced C2C12 myoblasts were expanded in R0K0 or R6K4 isotopically labelled DMEM (Dundee Cell Products) for at least six passages prior to differentiation. Myotubes were harvested in precipitation buffer (50 mM Tris–HCl pH 7.4, 50 mM NaCl, 1 mM EDTA, 1 mM EGTA, 10% glycerol, 0.5% NP40, 1 mM DTT, 50 mM NaF, 5 mM Na_4_P_2_O_7_, 1 mM Na_3_VO_4_, 0.5 mM PMSF, Calbiochem protease inhibitor cocktail), lysates incubated (4°C, 1 h) with GFP-trap beads (ChromoTek), precipitates washed four times with precipitation buffer and samples pooled prior to eluting, SDS–PAGE and Nano LC–MS/MS on an Orbitrap Velos (Thermo Fisher Scientific). Mass spectrometric detection and quantification was performed as previously published [39]. Shown is a subset of the interacting proteins identified.

### Immunoprecipitation and western blot analysis

Transduced C2C12 myotubes or Flp-In HEK293 cells transfected by polyethylenimine with GFP constructs for 48 h were harvested in precipitation buffer and GFP-trap performed as described above. For mouse muscle immunoprecipitations, anti-FLAG antibody was preconjugated to protein-G-coated agarose beads which were subsequently blocked in precipitation buffer containing 1% BSA and then incubated overnight at 4°C with homogenised quadriceps muscle (4 mg) or precipitation buffer and subsequently washed. For endogenous reciprocal immunoprecipitations, AMPK-α1 antibody or sheep IgG was preconjugated to protein-G-coated sepharose beads which were then incubated (4°C, 2 h) with lysates from C2C12 myotubes harvested in precipitation buffer and subsequently washed. Standard western blot procedures were performed. Signal was detected by either a LI-COR Odyssey infrared imaging system of fluorescently labelled secondary antibodies or ECL of HRP-conjugated secondary antibodies.

### Protein expression and recombinant interaction studies

Glutathione S-transferase (GST)-tagged or dual GST/His-tagged proteins were expressed upon IPTG induction in Escherichia coli BL21 (DE3) overnight at 15°C, purified using talon affinity resin (Clontech), eluted with 200 mM imidazole, 50 mM Tris–HCl (pH 8), 300 mM NaCl, 5 mM 2-mercaptoethanol, 5% glycerol and subsequently immobilised and purified on glutathione sepharose (GE Healthcare). For pull-down experiments, immobilised purified proteins (2 or 4 µg) were incubated (4°C, 30 min or 1 h) with Flp-In HEK293 cell lysate (5 mg) in precipitation buffer supplemented with 2 mM ATP and 5 mM MgCl_2_. Additional A769662 (25 µM) or vehicle was spiked into activation experiment pull-downs during the 4°C incubation step. After extensive washing bound proteins were detected via western blotting. Recombinant AMPK trimer complexes were expressed in *E. coli* and purified as previously published [40]. For direct interaction pull-down experiments, immobilised purified GST-PTB1 + 2 (2 µg) was incubated (4°C, 1 h) with the purified AMPK trimer complexes (1 µg) in supplemented precipitation buffer without EDTA and EGTA and handled as described above.

### Signalling experiments

Cells transfected for 48 h were treated as specified in figure legends and harvested in ice-cold lysis buffer (50 mM HEPES pH 7.4, 150 mM NaCl, 1% Triton-X-100, 1 mM Na_3_VO_4_, 30 mM NaF, 10 mM Na_4_P_2_O_7_, 10 mM EDTA, 0.5 mM PMSF, Calbiochem protease inhibitor cocktail). To reduce basal AMPK, cells were pre-treated with STO-609 (25 µM, 30 min) as previously described [41,42]. Protein content was determined by BCA assay (Thermo Fisher Scientific) and equal amounts were analysed.

### Statistics

GraphPad Prism 7.0 software was used for statistical analyses as indicated in the figure legends.

## Results

### AMPK, but not Akt, stably associates with TBC1D1

**Figure 1.**
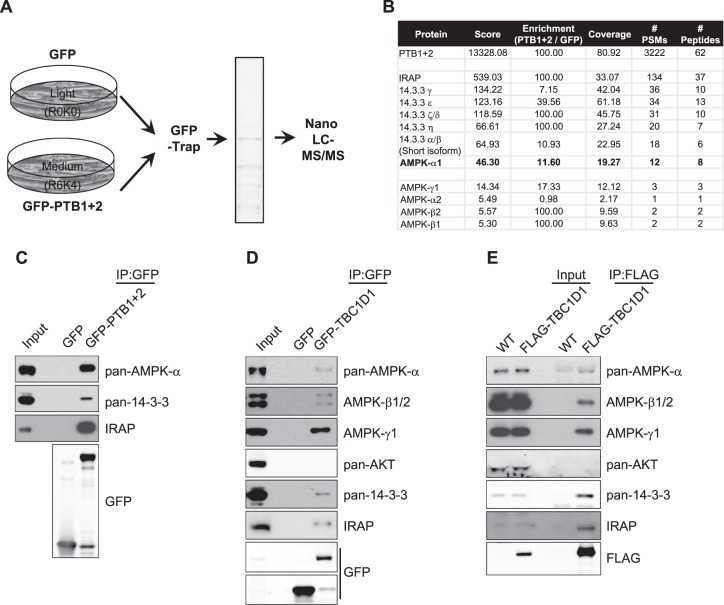
AMPK precipitates with TBC1D1 PTB domains in C2C12 myotubes. (**A**) Schematic of the SILAC experiment set-up used to identify proteins interacting with the PTB domains of TBC1D1 in differentiated C2C12 myotubes. (**B**) Table of a subset of the proteomics output. Score: combines several parameters (a high score generally signifies a high protein abundance and a high confidence in the detection and quantification by the software). Enrichment: Ratio of the quantification values of the medium (GFP-PTB 1 + 2) and light (GFP) quantification channels. Coverage: percentage of the protein sequence covered by identified peptides. # PSMs (peptide spectrum matches): total number of identified peptide sequences (includes those redundantly identified). # Peptides: total number of unique identified peptides. (**C**) Western blot validation of proteomics results. GFP-trap precipitates from C2C12 myotube extracts, lentivirally transduced to express either GFP or GFP-PTB1 + 2 blotted for the proteins indicated. (**D**) Lysates from differentiated C2C12 myotubes lentivirally transduced with GFP or full-length GFP-tagged TBC1D1 (GFP-TBC1D1) subjected to GFP-trap and western blotting for the proteins indicated. (**E**) Homogenised quadricep lysates from either wild-type (WT) mice or mice expressing 3xFLAG-TBC1D1 were immunoprecipitated with a FLAG antibody and resulting complexes analysed. Representative blots of *n* = 4 independent experiments.

In exploring the role of the PTB domains of TBC1D1, we used SILAC-based quantitative proteomics to identify proteins that interact with a GFP-tagged construct comprising the two tandem N-terminal PTB1-PTB2 domains (GFP-PTB1 + 2; Figure 1A). Immunoprecipitation via GFP-Trap from C2C12 myotubes stably expressing the GFP-PTB1 + 2 construct revealed several proteins to be 5- to 100-fold enriched in the GFP-PTB1 + 2 precipitates compared with the control (Figure 1B). This included protein families known to interact with the PTB domains of TBC1D1: IRAP (insulin-responsive aminopeptidase) and several isoforms of 14-3-3 proteins (the α/β, γ, ε, ζ/δ and η; Figure 1B) [15,21,24,25]. We also observed an 11.6-fold enrichment of the α1 subunit of AMPK, together with substantial enrichments of the β1, β2 and γ1 subunits (Figure 1B). This was validated by western blotting with a pan-α subunit antibody (Figure 1C). We additionally confirmed that the α, β and γ subunits of AMPK bound to full-length GFP-tagged TBC1D1 expressed in C2C12 myotubes (Figure 1D) and to a FLAG-tagged TBC1D1 transgene expressed in mouse quadriceps muscles (Figure 1E). These results indicate that AMPK heterotrimers bind to the TBC1D1 PTB domains with an affinity that is sufficiently high to survive immunoprecipitation and extensive washing.

Consistent with its absence in the SILAC data, we saw no evidence for a stable interaction of the Akt protein kinase with TBC1D1 expressed in either C2C12 myotubes or mouse quadriceps (Figure 1D,E). Given that both AMPK and Akt phosphorylate TBC1D1, albeit on different sites, our data suggest that the interaction of AMPK with TBC1D1 is not a general kinase-substrate-related phenomenon.

### TBC1D1 PTB domains directly and preferentially bind to AMPK complexes containing α1-subunits relative to α2-subunits

**Figure 2.**
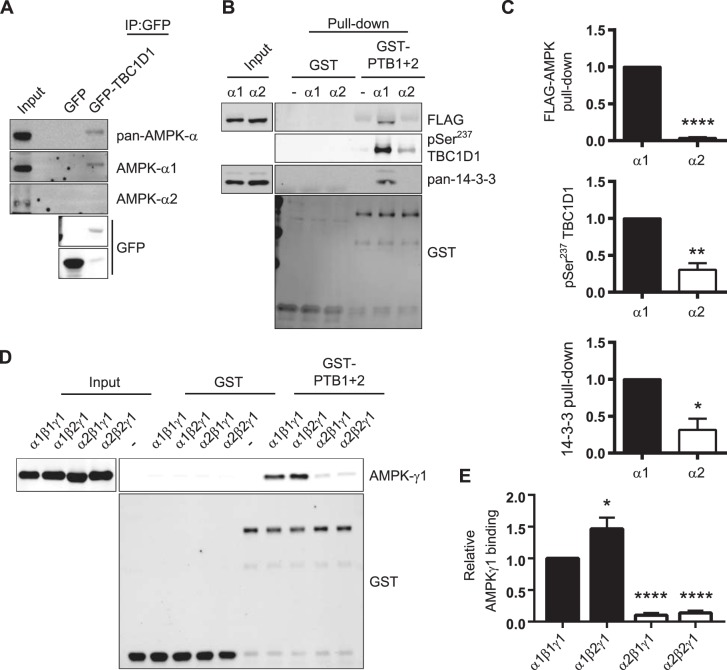
TBC1D1 binds AMPK-α1 and not AMPK-α2 heterotrimeric complexes. (**A**) Lysates from differentiated C2C12 myotubes lentivirally transduced with GFP or GFP-TBC1D1 subjected to GFP-trap and western blotting for AMPK-α isoforms. (**B**) Immobilised GST-His or GST-PTB1 + 2-His proteins were incubated with lysis buffer only (-) or lysates from Flp-In HEK293 cells stably expressing either FLAG-tagged AMPK-α1 or AMPK-α2. Washed complexes were analysed by western blotting. (**C**) Quantitation of data in (**B**), normalised to GST-PTB1 + 2-His and expressed in terms of FLAG-AMPK-α1 pull-down. Mean ± SEM; five independent experiments; two-tailed *t*-test *P < 0.05, **P < 0.01, ****P < 0.0001. (**D**) Immobilised GST or GST-PTB1 + 2 proteins were incubated with lysis buffer only (-) or purified recombinant AMPK αβγ heterotrimeric complexes as indicated and washed pull-downs analysed. Representative blots of four independent experiments. (**E**) Quantitation of data in (**D**), normalised to GST-PTB1 + 2. Mean ± SEM; four independent experiments; one-way ANOVA Dunnett's post-test *P < 0.05 ****P < 0.0001 *cf* pull-down of α1β1γ1.

Interestingly, our SILAC data revealed an enrichment of AMPK-α1 peptides in the TBC1D1 precipitates, but not of AMPK-α2 peptides (Figure 1B). Using isoform-specific AMPK-α antibodies [34], we confirmed that the interaction of TBC1D1 was indeed specific to AMPK complexes containing the α1 and not α2 subunit (Figure 2A).

To corroborate the selectivity in AMPK-α subunit binding, we incubated purified immobilised GST-PTB1 + 2 protein with lysates of Flp-In HEK293 cells stably expressing either FLAG-tagged AMPK-α1 or FLAG-tagged AMPK-α2 subunits [36]. Consistent with our previous results, GST-PTB1 + 2 interacted with FLAG-AMPK-α1 to a greater extent than with FLAG-AMPK-α2 (Figure 2B,C). Interestingly, phosphorylation of GST-PTB1 + 2 at Ser^237^, which occurred during the incubation of the recombinant protein with the lysate, was significantly higher in the presence of FLAG-AMPK-α1 relative to FLAG-AMPK-α2 and accordingly resulted in enhanced 14-3-3 protein pull-down (Figure 2B,C). Moreover, we performed the reciprocal immunoprecipitation to further confirm the interaction between FLAG-tagged AMPK-α1 and TBC1D1 (Supplementary Figure S1).

To assess whether the AMPK–TBC1D1 interaction was direct or indirect via another intermediary protein, such as 14-3-3, the purified immobilised GST-PTB1 + 2 protein was incubated with four different recombinant AMPK heterotrimer complexes purified from *E. coli*. Consistent with a direct interaction, AMPK heterotrimers comprising α1 subunits (namely α1β1γ1 and α1β2γ1) bound robustly, whereas those containing α2 subunits (α2β1γ1 and α2β2γ1) associated only weakly (Figure 2D,E). Taken together, the data indicate that AMPK heterotrimers comprising α1 but not α2 subunits bind directly to the PTB domains of TBC1D1.

### AMPK activation enhances the binding of AMPK-α1 to TBC1D1

**Figure 3.**
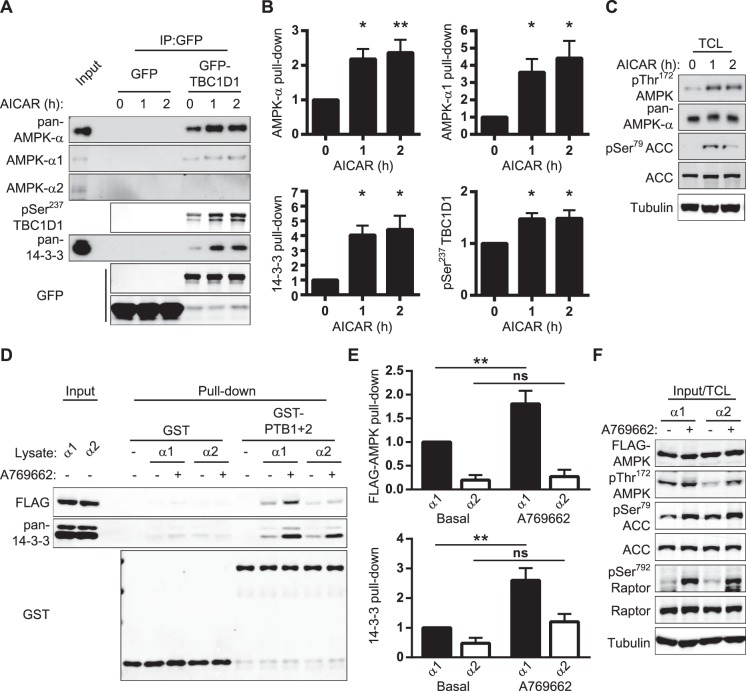
AMPK activation increases the association of AMPK-α1 with TBC1D1. (**A**) Western blot analysis of GFP-trap precipitates from differentiated C2C12 myotubes lentivirally transduced with GFP or GFP-TBC1D1, serum starved for 4–5 h, prior to the addition of vehicle or AICAR (2 mM) for the times indicated. (**B**) Quantitation of data shown in (**A**) normalised to GFP-TBC1D1 precipitated and presented as fold over basal pull-down. Mean ± SEM; three to four independent experiments; one-way ANOVA Dunnett's post-test *P < 0.05, **P < 0.01 *cf* Basal. (**C**) Total cell lysates (TCL) from (**A**) analysed for total and phosphorylated proteins. (**D**) Immobilised GST-His or GST-PTB1 + 2-His proteins were incubated with lysis buffer only (-) or lysates from Flp-In HEK293 cells stably expressing FLAG-AMPK-α1 or FLAG-AMPK-α2. Cells had been serum starved (4–5 h) prior to the addition of STO-609 (25 µM, 30 min) and subsequent treatment with either vehicle or A769662 (25 µM, 30 min). Washed complexes were analysed by western blotting. (**E**) Quantitation of data in (**D**), AMPK-α1 (black) and AMPK-α2 (white), normalised to GST-PTB1 + 2-His and expressed in terms of FLAG-tagged AMPK-α1 pull-down (black). Mean ± SEM; four to seven independent experiments; two-way ANOVA Bonferroni post-test ** *P* < 0.01. (**F**) Total cell lysates (TCL) from (**D**) analysed for total and phosphorylated proteins.

Next, we examined whether activation of AMPK regulates the interaction with TBC1D1. GFP-trap precipitates from C2C12 myotubes treated with the AMPK activator, AICAR, were analysed. As shown in Figure 3A,B, AICAR stimulated an ∼4-fold increase in the binding of TBC1D1 with AMPK complexes containing α1-subunits. This occurred together with parallel increases in TBC1D1 phosphorylation on Ser^237^ and 14-3-3 binding. The α2 subunit was not detected in precipitates (Figure 3A) despite pharmacological activation of AMPK that resulted in enhanced AMPK and ACC phosphorylation (Figure 3C). Likewise, co-immunoprecipitation of endogenous TBC1D1 with endogenous AMPK-α1 was increased upon AICAR stimulation in the C2C12 myotubes (Supplementary Figure S2).

A recent quantitative proteomic analysis reported significantly higher expression of the α1 subunit compared with α2 in C2C12 cells [43]. We thus sought to address whether the apparent lack of association with AMPK α2-subunits in the presence of an AMPK activator was not simply due to low endogenous α2 expression levels in these cells. To do this, we turned to the Flp-In HEK293 cell system which stably express equal levels of FLAG-AMPK-α1 or FLAG-AMPK-α2, largely replacing endogenous AMPK-α [36], and used the allosteric AMPK activator, A769662 (Figure 3D,E). Consistent with our previous results, A769662 promoted an increase in the binding of FLAG-AMPK-α1 but not of FLAG-AMPK-α2 to TBC1D1 PTB domains (Figure 3D,E) under conditions where AMPK was clearly activated as demonstrated by increases in AMPK, ACC and Raptor phosphorylation (Figure 3F).

### The AMPK–TBC1D1 interaction requires the TBC1D1 PTB2 domain is enhanced by the presence of the PTB1 domain and inhibited by a R125W mutation in PTB1

**Figure 4.**
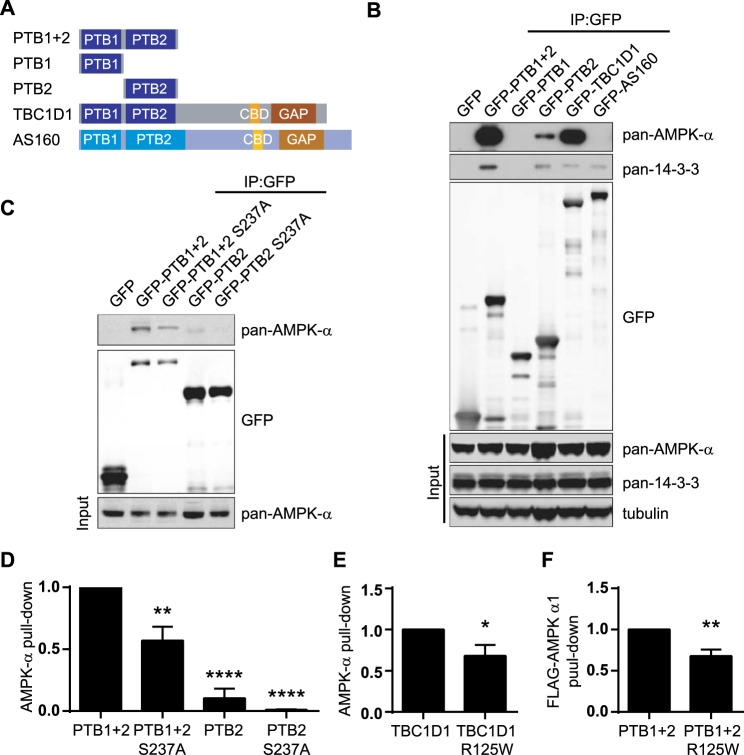
The AMPK–TBC1D1 interaction requires the TBC1D1 PTB2 domain is enhanced by the presence of the PTB1 domain and reduced by a R125W mutation in PTB1. (**A**) Schematic overview of the GFP-tagged constructs used in pull-down experiment in (**B**). (**B**) Lysates from differentiated C2C12 myotubes lentivirally transduced with GFP-tagged constructs as indicated were subjected to GFP-trap and western blotting for the proteins indicated. (**C**) Analysis of GFP-trap precipitates from differentiated C2C12 myotubes lentivirally transduced with indicated GFP-tagged constructs. (**D**) Quantitation of AMPK-α precipitation, shown in (**C**), normalised to GFP-tagged protein precipitated and expressed in terms of GFP-PTB1 + 2 pull-down. Mean ± SEM; three independent experiments; one-way ANOVA Dunnett's post-test **P < 0.01, ****P < 0.0001 *cf* GFP-PTB1 + 2. (**E**) Lysates from differentiated C2C12 myotubes lentivirally transduced with GFP-TBC1D1 or GFP-TBC1D1 R125W were subjected to GFP-trap and western blotting. Shown is the quantitation of AMPK-α subunit precipitated with wild-type or mutant protein normalised to GFP-tagged protein precipitated. Mean ± SEM; 12 independent experiments; two-tailed *t*-test *P < 0.05. (**F**) Immobilised recombinant purified GST-PTB1 + 2-His or GST-PTB1 + 2 R125W-His proteins were incubated with lysates from Flp-In HEK293 cells stably expressing FLAG-tagged AMPK-α1. Washed complexes were analysed by western blotting. Shown is the quantitation of FLAG-AMPK-α1 precipitated with wild-type or mutant protein normalised to GST-tagged protein content. Mean ± SEM; four independent experiments; two-tailed *t*-test **P < 0.01.

Our studies in the C2C12 myotube system revealed that AMPK-α subunits bound to the PTB2 domain, which contains the critical AMPK-directed Ser^237^phosphorylation site, but not to the PTB1 domain alone (Figure 4A,B). Interestingly, however, we consistently found that the presence of PTB1 enhanced the interaction as demonstrated by the greater pull-down with GFP-PTB1 + 2 and GFP-TBC1D1 compared with GFP-PTB2 (Figure 4B). This suggests there is co-operativity between the PTB1 and PTB2 domains in conferring optimal AMPK binding.

To explore the contribution of the enzyme/peptide substrate interaction interface (or ‘catalytic interface’) in facilitating the AMPK–TBC1D1 interaction, we mutated the AMPK-mediated phosphorylation site Ser^237^ to an alanine in the context of both PTB1 + 2 and PTB2 constructs (Figure 4C,D). Mutation of Ser^237^ abolished AMPK binding to a GFP-tagged PTB2 domain, suggesting the interaction of this domain with AMPK is dependent on the presence of a phosphorylatable residue. In contrast, however, the S237A mutation only partially (42%) inhibited AMPK binding to GFP-PTB1 + 2 (Figure 4C,D). Very similar results were obtained if we mutated Met^232^, Arg^233^ and Leu^241^ which are key consensus sequence residues surrounding Ser^237^ [44] (Supplementary Figure S3A,B). Importantly, while the S237A, M232A, R233A and L241A mutant forms of full-length TBC1D1 retained AMPK binding similar to the wild-type in the absence of A769662, the kinase was unable to phosphorylate Ser^237^ (Supplementary Figure S3B). We additionally found that the kinase activity of α1 was not required for TBC1D1 binding as, surprisingly, a kinase-dead mutant expressed in a CRISPR/Cas9 HEK293 α1/α2 double knockout (HEK293-DKO) cell line (Supplementary Figure S4A,B) showed enhanced binding to GST-PTB1 + 2 (Supplementary Figure S4C,D).

Taken together, these data suggest that the catalytic interface encompassing the Ser^237^ phosphorylation site is just one element of the mechanism involved in mediating the stable AMPK–TBC1D1 interaction, and that PTB1 plays an important synergistic role, perhaps by providing a second interaction interface with AMPK-α1.

Given the cooperation of the PTB1 domain with PTB2 in promoting the AMPKα1–TBC1D1 interaction, we considered whether the naturally occurring R125W mutation within the PTB1 domain, associated with a rare form of severe obesity, alters the interaction. The R125W mutation significantly reduced the association of AMPK with TBC1D1 in C2C12 myotubes (Figure 4E), an observation that was corroborated by reduced FLAG-AMPK-α1 pull-down, from Flp-in HEK293 cells, with GST-PTB1 + 2 R125W compared with the wild-type protein (Figure 4F). These data further implicate PTB1 in playing an important role in modifying the AMPK–TBC1D1 interaction.

### AMPK binds TBC1D1 but not AS160

We also investigated whether the ability of TBC1D1 to bind AMPK was shared by the paralogue AS160, which is highly expressed in adipose tissue but also found in both type I and II muscle fibres [45,46] with mouse glycolytic muscle previously shown to express a relatively lower level of AS160 [14]. TBC1D1 and AS160 possess similar domain structures (Figure 4A), exhibiting 37 and 43% sequence identity within their PTB1 and PTB2 domains, respectively [13]. Like TBC1D1, AS160 can be phosphorylated by AMPK on Ser^341^ within the PTB2 domain and on Ser^704^ [47,48]. However, unlike TBC1D1, AS160 did not appear to bind to AMPK in C2C12 myotubes under conditions where 14-3-3 proteins bound to both TBC1D1 and AS160 (Figure 4B).

### The stable association of AMPK promotes enhanced TBC1D1 Ser^237^ phosphorylation

**Figure 5.**
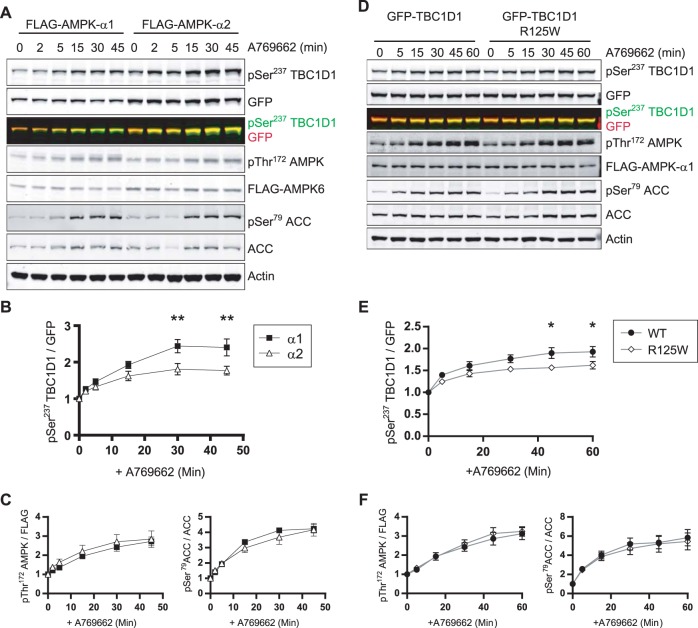
Cells expressing AMPK-α1 facilitate enhanced Ser237 phosphorylation of TBC1D1 which is reduced by the naturally occurring R125W mutation. (**A**) Analysis of phosphorylation of transiently expressed GFP-TBC1D1 as well as AMPK and ACC and total proteins from Flp-In HEK293 cells stably expressing FLAG-AMPK-α1 or FLAG-AMPK-α2. Cells had been serum starved (4–5 h) prior to the addition of STO-609 (25 µM, 30 min) and subsequent treatment with either vehicle or A769662 (25 µM) for the times indicated. (**B** and **C**) Quantitation of fluorescence-based Western blot data shown in (**A**) in FLAG-AMPK-α1 (black squares) and FLAG-AMPK-α2 (white triangles) expressing cells; Ser^237^ phosphorylation normalised to GFP-tagged protein expression; AMPK phosphorylation normalised to FLAG-AMPK-α1 or FLAG-AMPK-α2 expression; ACC phosphorylation normalised to total ACC expression. All displayed as a fold over basal phosphorylation. Mean ± SEM; six independent experiments; two-way ANOVA Bonferroni post-test **P < 0.01. (**D**) Quantitative fluorescence-based Western blot analysis of phosphorylation of TBC1D1, AMPK and ACC and total proteins from CRISPR/Cas9 α1/α2 double knockout (DKO) HEK293 stably expressing FLAG-AMPK-α1 and transiently expressing either GFP-TBC1D1 or GFP-TBC1D1 R125W. Cells had been serum starved (4–5 h) prior to the addition of STO-609 (25 µM, 30 min) and subsequent treatment with either vehicle or A769662 (25 µM) for the times indicated. (**E** and **F**) Quantitation of data shown in (**C**) in GFP-TBC1D1 (black circles) and GFP-TBC1D1 R125W (white diamonds) expressing cells; Ser^237^ phosphorylation normalised to GFP-tagged protein expression; AMPK phosphorylation normalised to FLAG-AMPK-α1 expression; ACC phosphorylation normalised to total ACC expression. All displayed as a fold over basal phosphorylation. Mean ± SEM; five independent experiments; two-way ANOVA Bonferroni post-test *P < 0.05.

To investigate whether the stable association of TBC1D1 with AMPK changes the kinetics of TBC1D1 phosphorylation at Ser^237^, we transiently expressed GFP-TBC1D1 in Flp-In HEK293 cells stably expressing FLAG-AMPK-α1 or FLAG-AMPK-α2. To reduce basal AMPK activation, cells were pre-treated with the Ca^2+^/calmodulin-dependent protein kinase kinase 2 inhibitor STO-609 as previously described [41,42]. As shown in Figure 5A,B, activation of AMPK with A769662 resulted in increased TBC1D1 Ser^237^ phosphorylation in both cell lines over the course of 45 min. However, cells expressing FLAG-AMPK-α1 subunits showed a significantly higher fold increase in TBC1D1 Ser^237^ phosphorylation than cells expressing FLAG-AMPKα2. This difference was specific to TBC1D1, since the phosphorylation of another substrate, ACC, as well as of AMPK on T172, was very similar in cells expressing FLAG-AMPK-α1 or FLAG-AMPK-α2 (Figure 5A,C). Taken together, this suggests that the stable association of AMPK-α1 with TBC1D1 which enhances the efficacy by which Ser^237^ is phosphorylated by AMPK.

The reduction in the AMPK–TBC1D1 interaction brought about by the R125W mutation would be predicted, based on our previous results (Figure 4E,F), to reduce TBC1D1 Ser^237^ phosphorylation. To explore this, we used the CRISPR/Cas9 HEK293-DKO cell line that stably expressed FLAG-AMPK-α1 (Supplementary Figure S4). A GFP-TBC1D1 R125W mutant transiently expressed in these cells did indeed exhibit reduced phosphorylation of Ser^237^ compared with the wild-type protein (Figure 5D,E). This was not due to a difference in cellular AMPK activity in the R125W expressing cells, as the kinetics of AMPK and ACC phosphorylation were the same in both cell lines (Figure 5D,F). Moreover, this was specific to cells expressing FLAG-AMPK-α1 as no difference in Ser^237^phosphorylation was observed in FLAG-AMPK-α2 expressing cells (Supplementary Figure S5).

## Discussion

Our findings demonstrate that AMPK heterotrimers containing α1 but not α2 subunits form a direct stable association with TBC1D1, but not with its paralogue AS160, which enhances the efficacy of phosphorylation of a TBC1D1 on Ser^237^, a key regulatory site. To the best of our knowledge, our results provide the first evidence for a substantive difference in substrate selection between the α1 and α2 subunits of AMPK *in vitro* or in intact cells; previous studies have revealed only very subtle differences [34]. The difference we observe is especially striking given the 76% sequence similarity between α1 and α2 subunits, which reaches 90% within the catalytic domains.

Typically, protein kinases do not bind substrates with sufficiently high affinity for their interaction to survive cell lysis, immunoprecipitation and extensive washing of the complexes. Based on our results, therefore, we propose a dual interaction mechanism for the stable interaction of AMPK α1-containing heterotrimers with TBC1D1.

One of these interaction mechanisms appears to involve the catalytic interface encompassing the substrate-binding cleft of the AMPK-α1 subunit and its target Ser^237^ phosphorylation site within the PTB2 domain. Evidence for this derives from the fact that the interaction: (i) is reduced by mutation of Ser^237^ and its neighbouring residues, Met^232^, Arg^233^ and Leu^241^, that together confer selective substrate binding by AMPK (Figure 4D and Supplementary Figure S3); and (ii) is enhanced by pharmacological AMPK activators (Figure 3). Furthermore, a kinase-inactive mutant of the AMPK-α1 subunit exhibits enhanced binding to TBC1D1 (Supplementary Figure S4), suggesting that the process of Ser^237^ phosphorylation may ultimately be required to release AMPK from TBC1D1.

We propose that a second interaction interface exists outside the substrate-binding cleft. Evidence for this derives from three observations: (i) that the presence of PTB1 substantially enhances the binding of the PTB2 domain to AMPK (Figure 4); (ii) that an R125W mutation in the PTB1 domain significantly reduces binding of TBC1D1 to AMPK (Figure 4E,F); and (iii) that disruption of the catalytic interface within PTB2 brought about by mutating Met^232^, Arg^233^, Ser^237^ or Leu^241^ only partially reduces the interaction between AMPK and TBC1D1 in the context of full-length TBC1D1 containing both PTB1 and PTB2 domains (Figure 4D and Supplementary Figure S3). These data suggest that the secondary binding interface could lie within the PTB1 domain, although if this is the case, the binding affinity is insufficient for the PTB1 domain to co-precipitate with AMPK when it is expressed in isolation (Figure 4B). Alternatively, the secondary binding interface could lie within the PTB2 domain and its affinity for AMPK-α1 subunits might be allosterically regulated by the presence of PTB1 or by the R125W mutation in the PTB1 domain. We cannot exclude a third possibility where regions within *both* the PTB1 and PTB2 domains contribute to the secondary binding interface.

Docking sites, external to protein kinase catalytic interfaces, are well established to facilitate alignment of the substrate phospho-acceptor site with its cognate protein kinase substrate-binding groove to enhance phosphorylation efficiency (reviewed in [49–51]). The best-characterised examples of this include a MAP kinase docking site which can drive both positive and negative substrate selections, and PDK1 which possesses a hydrophobic pocket that interacts with a hydrophobic motif present on all its substrates [49]. Formal identification of the mode of binding between AMPK and TBC1D1 will require structural studies of the purified complex.

It is well established that muscle contraction stimulates AMPK activity and increases TBC1D1 Ser^237^ phosphorylation and that this is associated with an increase in muscle glucose uptake [52]. Our data support a role for the stable interaction between AMPK-α1 heterotrimers and TBC1D1 in enhancing TBC1D1 Ser^237^ phosphorylation. This is substantiated by our observations that TBC1D1 Ser^237^ phosphorylation is: (i) enhanced in cells expressing α1 subunit-containing AMPK heterotrimers versus those expressing α2-containing heterotrimers (under conditions where phosphorylation of ACC on Ser^79^ was no different between AMPK-α1 and AMPK-α2 heterotrimeric expressing cells; Figure 5); and (ii) is reduced in cells expressing the R125W mutant TBC1D1 versus those expressing the wild-type protein (Figure 5). The observed alteration in phosphorylation of Ser^237^ could have important downstream signalling consequences. For example, while insulin does not regulate TBC1D1 Ser^237^ phosphorylation, our data could explain the previously reported ability of the R125W mutation in TBC1D1 to prevent insulin-stimulated trafficking of GLUT4 to the plasma membrane, following a prerequisite AICAR pre-stimulation, when exogenously expressed in pre-adipocytes [23] as well as inhibiting insulin-stimulated, but interestingly not electrically induced contraction-stimulated, glucose transport when overexpressed in mouse skeletal muscle [53]. These observations could be related to inhibition of insulin-stimulated glucose uptake by the binding of APPL2 to TBC1D1 at an adjacent insulin-stimulated Ser^235^ phosphorylation site [54]. Given the proximity of this binding site to Ser^237^, the association of TBC1D1 and APPL2 may be affected by the physical presence of AMPK and thus it is conceivable that reduced AMPK binding to TBC1D1 R125W may facilitate APPL2-mediated inhibition of insulin-stimulated glucose transport. Unfortunately, we have not been able to test this hypothesis because we have been unable to detect the binding of APPL2 to TBC1D1 in C2C12 myotubes.

The roles of α1- and α2-containing AMPK heterotrimers in tissues are the subject of much debate in the literature. AMPK complexes containing α2, but not α1, subunits are reported to be activated by contraction [55–59] and that contraction-induced phosphorylation of Ser^237^ phosphorylation is impaired in α2, but not α1, knockout mice [24]. In mouse models where AMPK-α2 is either inactivated or knocked-out in skeletal muscle, contraction-stimulated glucose uptake remains normal [6,7,60] or only partially reduced [5,8], whereas it is substantially impaired in β1/β2 knockout muscles, where all AMPK kinase activity is completely lost [61]. The latter being congruent with other groups who have suggested that AMPK-α1 and AMPK-α2 heterotrimeric complexes respond to specific types and durations of contraction making the picture much more complicated [62–68].

AMPK has been proposed as an attractive drug target for the treatment of metabolic diseases which has led to great interest in the development of isoform selective compounds [69], such the allosteric activator of α1-containing heterotrimeric complexes C2 [70]. While the differential roles of the catalytic α-subunit are currently limited [3] distinctions between the catalytic subunits, such as the association of α1 subunit-containing AMPK heterotrimers with TBC1D1, may enable selective modification of AMPK function.

The binding of α1-containing complexes to TBC1D1 may have important roles in non-muscle cell types where α1 subunit expression and activity predominates over α2. In human hepatocytes, for example, expression of α1β2γ1 heterotrimers predominates [71], and in these cells metformin stimulates Ser^237^ phosphorylation on TBC1D1 via AMPK activation [19]. Interestingly, in mouse hepatocytes TBC1D1 localises to insulin-like growth factor 1 (IGF1)-containing storage vesicles, and a TBC1D1 S231A (equivalent to Ser^237^ in humans) knock-in mutation promotes increased IGF1 secretion from the liver, leading to lipogenic gene expression in adipose tissue and consequent obesity [19]. Taken together with our data, this could suggest that in hepatocytes the α1β2γ1heterotrimer stably bound to TBC1D1 plays an important role in metformin-induced suppression of IGF1 secretion, a phenomenon that is reduced in the S231A knock-in mouse. Moreover, given the (i) reduced Ser^237^ phosphorylation of the R125W mutant demonstrated in our experiments and (ii) IGF1 hypersecretion-mediated obesity in the knock-in mouse where Ser^231^ can no longer be phosphorylated; an attractive hypothesis is that the observed severe obesity phenotype in a subset of women with the R125W mutation may occur via a mechanism involving elevated circulating levels of IGF1. In summary, we have shown that α1-containing AMPK heterotrimers bind stably to TBC1D1 via its PTB domains. This interaction plays an important role in controlling the degree of Ser^237^ phosphorylation on TBC1D1 and is thus likely to be important in controlling energy homeostasis in tissues co-expressing α1-containing AMPK heterotrimers and TBC1D1.

## Abbreviations

ACC: acetyl-CoA carboxylase
AICAR: 5-aminoimidazole-4-carboxamide riboside
AMPK: AMP-activated protein kinase
DKO: double knockout
GAP: GTPase-activating protein
GST: glutathione S-transferase
IGF1: insulin-like growth factor 1
PTB: phosphotyrosine binding
SILAC: Stable Isotope Labelling by Amino acids in Cell

## References

[1] Hardie D.G. (2014) AMPK — sensing energy while talking to other signaling pathways. Cell Metab. 20, 939–952 10.1016/j.cmet.2014.09.013 25448702 PMC5693325

[2] Carling D. (2017) AMPK signalling in health and disease. Curr. Opin. Cell Biol. 45, 31–37 10.1016/j.ceb.2017.01.005 28232179

[3] Ross F.A., MacKintosh C. and Hardie D.G. (2016) AMP-activated protein kinase: a cellular energy sensor that comes in 12 flavours. FEBS J. 283, 2987–3001 10.1111/febs.13698 26934201 PMC4995730

[4] Hawley S.A., Davison M., Woods A., Davies S.P., Beri R.K., Carling D. et al. (1996) Characterization of the AMP-activated protein kinase kinase from rat liver and identification of threonine 172 as the major site at which it phosphorylates AMP-activated protein kinase. J. Biol. Chem. 271, 27879–27887 10.1074/jbc.271.44.27879 8910387

[5] Lefort N., St-Amand E., Morasse S., Cote C.H. and Marette A. (2008) The α-subunit of AMPK is essential for submaximal contraction-mediated glucose transport in skeletal muscle in vitro. Am. J. Physiol. Endocrinol. Metab. 295, E1447–E1454 10.1152/ajpendo.90362.2008 18812461

[6] Jorgensen S.B., Viollet B., Andreelli F., Frosig C., Birk J.B., Schjerling P. et al. (2004) Knockout of the α2 but not α_1_ 5′-AMP-activated protein kinase isoform abolishes 5-aminoimidazole-4-carboxamide-1-β-4-ribofuranosidebut not contraction-induced glucose uptake in skeletal muscle. J. Biol. Chem. 279, 1070–1079 10.1074/jbc.M306205200 14573616

[7] Fujii N., Hirshman M.F., Kane E.M., Ho R.C., Peter L.E., Seifert M.M. et al. (2005) AMP-activated protein kinase α2 activity is not essential for contraction- and hyperosmolarity-induced glucose transport in skeletal muscle. J. Biol. Chem. 280, 39033–39041 10.1074/jbc.M504208200 16186119

[8] Mu J., Brozinick J.T., Jr. Valladares O., Bucan M. and Birnbaum M.J. (2001) A role for AMP-activated protein kinase in contraction- and hypoxia-regulated glucose transport in skeletal muscle. Mol. Cell 7, 1085–1094 10.1016/S1097-2765(01)00251-9 11389854

[9] Merrill G.F., Kurth E.J., Hardie D.G. and Winder W.W. (1997) AICA riboside increases AMP-activated protein kinase, fatty acid oxidation, and glucose uptake in rat muscle. Am. J. Physiol. 273(6 Pt 1), E1107–E1112. 9435525 10.1152/ajpendo.1997.273.6.E1107

[10] Kurth-Kraczek E.J., Hirshman M.F., Goodyear L.J. and Winder W.W. (1999) 5′ AMP-activated protein kinase activation causes GLUT4 translocation in skeletal muscle. Diabetes 48, 1667–1671 10.2337/diabetes.48.8.1667 10426389

[11] Peck G.R., Chavez J.A., Roach W.G., Budnik B.A., Lane W.S., Karlsson H.K. et al. (2009) Insulin-stimulated phosphorylation of the Rab GTPase-activating protein TBC1D1 regulates GLUT4 translocation. J. Biol. Chem. 284, 30016–30023 10.1074/jbc.M109.035568 19740738 PMC2781555

[12] Chadt A., Leicht K., Deshmukh A., Jiang L.Q., Scherneck S., Bernhardt U. et al. (2008) Tbc1d1 mutation in lean mouse strain confers leanness and protects from diet-induced obesity. Nat. Genet. 40, 1354–1359 10.1038/ng.244 18931681

[13] Roach W.G., Chavez J.A., Mîinea C.P. and Lienhard G.E. (2007) Substrate specificity and effect on GLUT4 translocation of the Rab GTPase-activating protein Tbc1d1. Biochem. J. 403, 353–358 10.1042/BJ20061798 17274760 PMC1874243

[14] Taylor E.B., An D., Kramer H.F., Yu H., Fujii N.L., Roeckl K.S. et al. (2008) Discovery of TBC1D1 as an insulin-, AICAR-, and contraction-stimulated signaling nexus in mouse skeletal muscle. J. Biol. Chem. 283, 9787–9796 10.1074/jbc.M708839200 18276596 PMC2442306

[15] Pehmoller C., Treebak J.T., Birk J.B., Chen S., Mackintosh C., Hardie D.G. et al. (2009) Genetic disruption of AMPK signaling abolishes both contraction- and insulin-stimulated TBC1D1 phosphorylation and 14-3-3 binding in mouse skeletal muscle. Am. J. Physiol. Endocrinol. Metab. 297, E665–E675 10.1152/ajpendo.00115.2009 19531644 PMC2739697

[16] Szekeres F., Chadt A., Tom R.Z., Deshmukh A.S., Chibalin A.V., Bjornholm M. et al. (2012) The Rab-GTPase-activating protein TBC1D1 regulates skeletal muscle glucose metabolism. Am. J. Physiol. Endocrinol. Metab. 303, E524–E533 10.1152/ajpendo.00605.2011 22693207

[17] Kosfeld A., Kreuzer M., Daniel C., Brand F., Schafer A.K., Chadt A. et al. (2016) Whole-exome sequencing identifies mutations of TBC1D1 encoding a Rab-GTPase-activating protein in patients with congenital anomalies of the kidneys and urinary tract (CAKUT). Hum. Genet. 135, 69–87 10.1007/s00439-015-1610-1 26572137

[18] Rütti S., Arous C., Nica A.C., Kanzaki M., Halban P.A. and Bouzakri K. (2014) Expression, phosphorylation and function of the Rab-GTPase activating protein TBC1D1 in pancreatic beta-cells. FEBS Lett. 588, 15–20 10.1016/j.febslet.2013.10.050 24239544

[19] Chen L., Chen Q., Xie B., Quan C., Sheng Y., Zhu S. et al. (2016) Disruption of the AMPK-TBC1D1 nexus increases lipogenic gene expression and causes obesity in mice via promoting IGF1 secretion. Proc. Natl Acad. Sci. U.S.A. 113, 7219–7224 10.1073/pnas.1600581113 27307439 PMC4932950

[20] Stone S., Abkevich V., Russell D.L., Riley R., Timms K., Tran T. et al. (2006) TBC1D1 is a candidate for a severe obesity gene and evidence for a gene/gene interaction in obesity predisposition. Hum. Mol. Genet. 15, 2709–2720 10.1093/hmg/ddl204 16893906

[21] Tan S.X., Ng Y., Burchfield J.G., Ramm G., Lambright D.G., Stockli J. et al. (2012) The Rab GTPase-activating protein TBC1D4/AS160 contains an atypical phosphotyrosine-binding domain that interacts with plasma membrane phospholipids to facilitate GLUT4 trafficking in adipocytes. Mol. Cell Biol. 32, 4946–4959 10.1128/MCB.00761-12 23045393 PMC3510527

[22] Koumanov F., Richardson J.D., Murrow B.A. and Holman G.D. (2011) AS160 phosphotyrosine-binding domain constructs inhibit insulin-stimulated GLUT4 vesicle fusion with the plasma membrane. J. Biol. Chem. 286, 16574–16582 10.1074/jbc.M111.226092 21454690 PMC3089500

[23] Hatakeyama H. and Kanzaki M. (2013) Regulatory mode shift of Tbc1d1 is required for acquisition of insulin-responsive GLUT4-trafficking activity. Mol. Biol. Cell 24, 809–817 10.1091/mbc.e12-10-0725 23325788 PMC3596251

[24] Frosig C., Pehmoller C., Birk J.B., Richter E.A. and Wojtaszewski J.F. (2010) Exercise-induced TBC1D1 Ser237 phosphorylation and 14-3-3 protein binding capacity in human skeletal muscle. J. Physiol. 588(Pt 22), 4539–4548 10.1113/jphysiol.2010.194811 20837646 PMC3008856

[25] Chen S., Murphy J., Toth R., Campbell D.G., Morrice N.A. and Mackintosh C. (2008) Complementary regulation of TBC1D1 and AS160 by growth factors, insulin and AMPK activators. Biochem. J. 409, 449–459 10.1042/BJ20071114 17995453

[26] Rubin C.J., Zody M.C., Eriksson J., Meadows J.R., Sherwood E., Webster M.T. et al. (2010) Whole-genome resequencing reveals loci under selection during chicken domestication. Nature 464, 587–591 10.1038/nature08832 20220755

[27] Fontanesi L., Colombo M., Tognazzi L., Scotti E., Buttazzoni L., Dall'Olio S. et al. (2011) The porcine TBC1D1 gene: mapping, SNP identification, and association study with meat, carcass and production traits in Italian heavy pigs. Mol. Biol. Rep. 38, 1425–1431 10.1007/s11033-010-0247-3 20730498

[28] Fontanesi L., Galimberti G., Calo D.G., Fronza R., Martelli P.L., Scotti E. et al. (2012) Identification and association analysis of several hundred single nucleotide polymorphisms within candidate genes for back fat thickness in Italian Large White pigs using a selective genotyping approach. J. Anim. Sci. 90, 2450–2464 PMID:22367074 10.2527/jas.2011-4797

[29] Yang Z., Fu L., Zhang G., Yang Y., Chen S., Wang J. et al. (2013) Identification and association of SNPs in TBC1D1 gene with growth traits in two rabbit breeds. Asian-Australas. J. Anim. Sci. 26, 1529–1535 10.5713/ajas.2013.13278 25049738 PMC4093812

[30] Fox C.S., Heard-Costa N., Cupples L.A., Dupuis J., Vasan R.S. and Atwood L.D. (2007) Genome-wide association to body mass index and waist circumference: the Framingham Heart Study 100K project. BMC Med. Genet. 8, S18 10.1186/1471-2350-8-S1-S18 17903300 PMC1995618

[31] Knuppel S., Rohde K., Meidtner K., Drogan D., Holzhutter H.G., Boeing H. et al. (2013) Evaluation of 41 candidate gene variants for obesity in the EPIC-potsdam cohort by multi-locus stepwise regression. PLoS ONE 8, e68941 10.1371/journal.pone.0068941 23874820 PMC3709896

[32] Meyre D., Farge M., Lecoeur C., Proenca C., Durand E., Allegaert F. et al. (2008) R125W coding variant in TBC1D1 confers risk for familial obesity and contributes to linkage on chromosome 4p14 in the French population. Hum. Mol. Genet. 17, 1798–1802 10.1093/hmg/ddn070 18325908

[33] Richardson T.G., Thomas E.C., Sessions R.B., Lawlor D.A., Tavare J.M. and Day I.N. (2013) Structural and population-based evaluations of TBC1D1 p.Arg125Trp. PLoS ONE 8, e63897 10.1371/journal.pone.0063897 23667688 PMC3646766

[34] Woods A., Salt I., Scott J., Hardie D.G. and Carling D. (1996) The α1 and α2 isoforms of the AMP-activated protein kinase have similar activities in rat liver but exhibit differences in substrate specificity in vitro. FEBS Lett. 397, 347–351 10.1016/S0014-5793(96)01209-4 8955377

[35] Nedachi T., Fujita H. and Kanzaki M. (2008) Contractile C2C12 myotube model for studying exercise-inducible responses in skeletal muscle. Am. J. Physiol. Endocrinol. Metab. 295, E1191–E1204 10.1152/ajpendo.90280.2008 18780777

[36] Hawley S.A., Ross F.A., Gowans G.J., Tibarewal P., Leslie N.R. and Hardie D.G. (2014) Phosphorylation by Akt within the ST loop of AMPK-α1 down-regulates its activation in tumour cells. Biochem. J. 459, 275–287 10.1042/BJ20131344 24467442 PMC4052680

[37] Ran F.A., Hsu P.D., Wright J., Agarwala V., Scott D.A. and Zhang F. (2013) Genome engineering using the CRISPR-Cas9 system. Nat. Protoc. 8, 2281–2308 10.1038/nprot.2013.143 24157548 PMC3969860

[38] Fogarty S., Ross F.A., Vara Ciruelos D., Gray A., Gowans G.J. and Hardie D.G. (2016) AMPK causes cell cycle arrest in LKB1-deficient cells via activation of CAMKK2. Mol. Cancer Res. 14, 683–695 10.1158/1541-7786.MCR-15-0479 27141100 PMC5390849

[39] Steinberg F., Heesom K.J., Bass M.D. and Cullen P.J. (2012) SNX17 protects integrins from degradation by sorting between lysosomal and recycling pathways. J. Cell Biol. 197, 219–230 10.1083/jcb.201111121 22492727 PMC3328392

[40] Xiao B., Sanders M.J., Carmena D., Bright N.J., Haire L.F., Underwood E. et al. (2013) Structural basis of AMPK regulation by small molecule activators. Nat. Commun. 4, 3017 10.1038/ncomms4017 24352254 PMC3905731

[41] Vincent E.E., Coelho P.P., Blagih J., Griss T., Viollet B. and Jones R.G. (2015) Differential effects of AMPK agonists on cell growth and metabolism. Oncogene 34, 3627–3639 10.1038/onc.2014.301 25241895 PMC4980123

[42] Gwinn D.M., Shackelford D.B., Egan D.F., Mihaylova M.M., Mery A., Vasquez D.S. et al. (2008) AMPK phosphorylation of raptor mediates a metabolic checkpoint. Mol. Cell 30, 214–226 10.1016/j.molcel.2008.03.003 18439900 PMC2674027

[43] Deshmukh A.S., Murgia M., Nagaraj N., Treebak J.T., Cox J. and Mann M. (2015) Deep proteomics of mouse skeletal muscle enables quantitation of protein isoforms, metabolic pathways and transcription factors. Mol. Cell. Proteomics 14, 841–853 10.1074/mcp.M114.044222 25616865 PMC4390264

[44] Hardie D.G. (2011) AMP-activated protein kinase — an energy sensor that regulates all aspects of cell function. Genes Dev. 25, 1895–1908 10.1101/gad.17420111 21937710 PMC3185962

[45] Castorena C.M., Mackrell J.G., Bogan J.S., Kanzaki M. and Cartee G.D. (2011) Clustering of GLUT4, TUG, and RUVBL2 protein levels correlate with myosin heavy chain isoform pattern in skeletal muscles, but AS160 and TBC1D1 levels do not. J. Appl. Physiol. 111, 1106–1117 10.1152/japplphysiol.00631.2011 21799128 PMC3191788

[46] Albers P.H., Pedersen A.J., Birk J.B., Kristensen D.E., Vind B.F., Baba O. et al. (2015) Human muscle fiber type-specific insulin signaling: impact of obesity and type 2 diabetes. Diabetes 64, 485–497 10.2337/db14-0590 25187364

[47] Treebak J.T., Taylor E.B., Witczak C.A., An D., Toyoda T., Koh H.J. et al. (2010) Identification of a novel phosphorylation site on TBC1D4 regulated by AMP-activated protein kinase in skeletal muscle. Am. J. Physiol. Cell Physiol. 298, C377–C385 10.1152/ajpcell.00297.2009 19923418 PMC2822490

[48] Treebak J.T., Pehmoller C., Kristensen J.M., Kjobsted R., Birk J.B., Schjerling P. et al. (2014) Acute exercise and physiological insulin induce distinct phosphorylation signatures on TBC1D1 and TBC1D4 proteins in human skeletal muscle. J. Physiol. 592, 351–375 10.1113/jphysiol.2013.266338 24247980 PMC3922499

[49] Biondi R.M. and Nebreda A.R. (2003) Signalling specificity of Ser/Thr protein kinases through docking-site-mediated interactions. Biochem. J. 372, 1–13 10.1042/bj20021641 12600273 PMC1223382

[50] Holland P.M. and Cooper J.A. (1999) Protein modification: docking sites for kinases. Curr. Biol. 9, R329–R331 10.1016/S0960-9822(99)80205-X 10322109

[51] Miller C.J. and Turk B.E. (2018) Homing in: mechanisms of substrate targeting by protein kinases. Trends Biochem. Sci. 43, 380–394 10.1016/j.tibs.2018.02.009 29544874 PMC5923429

[52] Cartee G.D. (2015) Roles of TBC1D1 and TBC1D4 in insulin- and exercise-stimulated glucose transport of skeletal muscle. Diabetologia 58, 19–30 10.1007/s00125-014-3395-5 25280670 PMC4258142

[53] An D., Toyoda T., Taylor E.B., Yu H., Fujii N., Hirshman M.F. et al. (2010) TBC1D1 regulates insulin- and contraction-induced glucose transport in mouse skeletal muscle. Diabetes 59, 1358–1365 10.2337/db09-1266 20299473 PMC2874696

[54] Cheng K.K., Zhu W., Chen B., Wang Y., Wu D., Sweeney G. et al. (2014) The adaptor protein APPL2 inhibits insulin-stimulated glucose uptake by interacting with TBC1D1 in skeletal muscle. Diabetes 63, 3748–3758 10.2337/db14-0337 24879834

[55] Hayashi T., Hirshman M.F., Fujii N., Habinowski S.A., Witters L.A. and Goodyear L.J. (2000) Metabolic stress and altered glucose transport: activation of AMP-activated protein kinase as a unifying coupling mechanism. Diabetes 49, 527–531 10.2337/diabetes.49.4.527 10871188

[56] Wojtaszewski J.F., Nielsen P., Hansen B.F., Richter E.A. and Kiens B. (2000) Isoform-specific and exercise intensity-dependent activation of 5′-AMP-activated protein kinase in human skeletal muscle. J. Physiol. 528, 221–226 10.1111/j.1469-7793.2000.t01-1-00221.x 11018120 PMC2270117

[57] Nielsen JN, Mustard KJ, Graham DA, Yu H, MacDonald CS, Pilegaard H, et al 5′-AMP-activated protein kinase activity and subunit expression in exercise-trained human skeletal muscle. J. Appl. Physiol. 2003;94:631-641. 10.1152/japplphysiol.00642.2002 12391032

[58] Yu M., Stepto N.K., Chibalin A.V., Fryer L.G., Carling D., Krook A. et al. (2003) Metabolic and mitogenic signal transduction in human skeletal muscle after intense cycling exercise. J. Physiol. 546, 327–335 10.1113/jphysiol.2002.034223 12527721 PMC2342514

[59] Wojtaszewski J.F., MacDonald C., Nielsen J.N., Hellsten Y., Hardie D.G., Kemp B.E. et al. (2003) Regulation of 5′AMP-activated protein kinase activity and substrate utilization in exercising human skeletal muscle. Am. J. Physiol. Endocrinol. Metab. 284, E813–E822 10.1152/ajpendo.00436.2002 12488245

[60] Merry T.L., Steinberg G.R., Lynch G.S. and McConell G.K. (2010) Skeletal muscle glucose uptake during contraction is regulated by nitric oxide and ROS independently of AMPK. Am. J. Physiol. Endocrinol. Metab. 298, E577–E585 10.1152/ajpendo.00239.2009 20009026

[61] O'Neill H.M., Maarbjerg S.J., Crane J.D., Jeppesen J., Jorgensen S.B., Schertzer J.D. et al. (2011) AMP-activated protein kinase (AMPK) β1β2 muscle null mice reveal an essential role for AMPK in maintaining mitochondrial content and glucose uptake during exercise. Proc. Natl Acad. Sci. U.S.A. 108, 16092–7 10.1073/pnas.1105062108 21896769 PMC3179037

[62] Chen Z.P., McConell G.K., Michell B.J., Snow R.J., Canny B.J. and Kemp B.E. (2000) AMPK signaling in contracting human skeletal muscle: acetyl-CoA carboxylase and NO synthase phosphorylation. Am. J. Physiol. Endocrinol. Metab. 279, E1202–6 10.1152/ajpendo.2000.279.5.E1202 11052978

[63] Chen Z.P., Stephens T.J., Murthy S., Canny B.J., Hargreaves M., Witters L.A. et al. (2003) Effect of exercise intensity on skeletal muscle AMPK signaling in humans. Diabetes 52, 2205–2212 10.2337/diabetes.52.9.2205 12941758

[64] McConell G.K., Lee-Young R.S., Chen Z.P., Stepto N.K., Huynh N.N., Stephens T.J. et al. (2005) Short-term exercise training in humans reduces AMPK signalling during prolonged exercise independent of muscle glycogen. J. Physiol. 568, 665–676 10.1113/jphysiol.2005.089839 16051629 PMC1474728

[65] Frosig C., Jorgensen S.B., Hardie D.G., Richter E.A. and Wojtaszewski J.F. (2004) 5′-AMP-activated protein kinase activity and protein expression are regulated by endurance training in human skeletal muscle. Am. J. Physiol. Endocrinol. Metab. 286, E411–E417 10.1152/ajpendo.00317.2003 14613924

[66] Lee-Young R.S., Palmer M.J., Linden K.C., LePlastrier K., Canny B.J., Hargreaves M. et al. (2006) Carbohydrate ingestion does not alter skeletal muscle AMPK signaling during exercise in humans. Am. J. Physiol. Endocrinol. Metab. 291, E566–E573 10.1152/ajpendo.00023.2006 16670154

[67] Toyoda T., Tanaka S., Ebihara K., Masuzaki H., Hosoda K., Sato K. et al. (2006) Low-intensity contraction activates the α1-isoform of 5′-AMP-activated protein kinase in rat skeletal muscle. Am. J. Physiol. Endocrinol. Metab. 290, E583–E590 10.1152/ajpendo.00395.2005 16249251

[68] Jensen T.E., Schjerling P., Viollet B., Wojtaszewski J.F. and Richter E.A. (2008) AMPK α1 activation is required for stimulation of glucose uptake by twitch contraction, but not by H_2_O_2_, in mouse skeletal muscle. PLoS ONE 3, e2102 10.1371/journal.pone.0002102 18461163 PMC2346549

[69] Olivier S., Foretz M. and Viollet B. (2018) Promise and challenges for direct small molecule AMPK activators. Biochem. Pharmacol. 153, 147–158 10.1016/j.bcp.2018.01.049 29408352

[70] Hunter R.W., Foretz M., Bultot L., Fullerton M.D., Deak M., Ross F.A. et al. (2014) Mechanism of action of compound-13: an α1-selective small molecule activator of AMPK. Chem. Biol. 21, 866–879 10.1016/j.chembiol.2014.05.014 25036776 PMC4104029

[71] Wu J., Puppala D., Feng X., Monetti M., Lapworth A.L. and Geoghegan K.F. (2013) Chemoproteomic analysis of intertissue and interspecies isoform diversity of AMP-activated protein kinase (AMPK). J. Biol. Chem. 288, 35904–35912 10.1074/jbc.M113.508747 24187138 PMC3861640

