# Supplementary material for: Isoform-specific AMPK association with TBC1D1 is reduced by a mutation associated with severe obesity
Source: Biochem J. 2018 Sep 25;475(18):2969–83. doi: 10.1042/BCJ20180475 (PMC6156765; doi:10.1042/BCJ20180475)

SUPPLEMENTARY DATA

Figure S1. Reciprocal binding of TBC1D1 to immunoprecipitated AMPK-α1.
Western blot analysis of Flp-In HEK293 cells expressing FLAG-tagged AMPK-α1 and indicated GFP-tagged proteins immunoprecipitated with a FLAG antibody. Representative of 2 independent experiments.

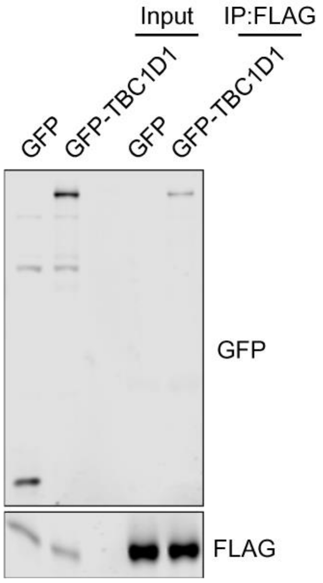

Figure S2. Reciprocal binding of endogenous TBC1D1 to endogenous AMPK-α1. (A) Lysates from differentiated C2C12 myotubes serum starved for 4-5 h prior to addition of vehicle or AICAR (2 mM, 2 h) were immunoprecipitated with the AMPK-α1 isoform specific antibody or IgG control and washed complexes analysed for the presence of endogenous TBC1D1. (B) Quantitation of data in A, normalised to AMPK-α pull-down and expressed in terms of basal. Mean ± SEM; 3 independent experiments; two-tailed t-test **p<0.01.

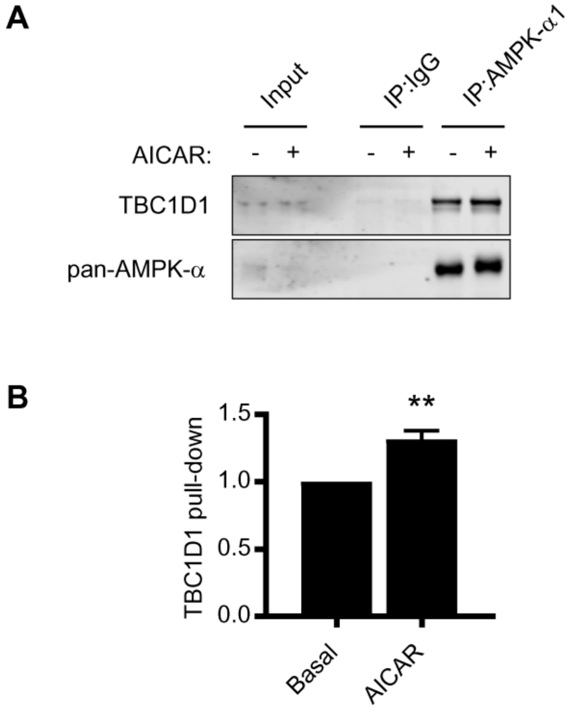

Figure S3. AMPK-α1 binding to TBC1D1 is partially dependent on the presence of an intact AMPK consensus motif. (A) AMPK substrate consensus motif sequence aligned with the TBC1D1 sequence surrounding Ser[237]. Hydrophobic residues are indicated by Φ and basic residues are shown as β. (B) Quantification of western blot analysis of GFP trap precipitates from Flp-In HEK293 cells stably expressing FLAG-AMPK-α1 transiently expressing full-length GFP-TBC1D1 with the indicated mutations. Cells were serum starved for 4-5 h prior to addition of STO-609 (25 μM, 30 min) followed by treatment with either vehicle or A769662 (25 μM) for 30 min. Samples were normalised to GFP-TBC1D1 precipitated and expressed in terms of the WT basal condition. Mean ± SEM; 4 independent experiments; one-way ANOVA Dunnett's post-test * $p<0.05$ **$p<0.01$, ***$p<0.001$ cf WT with A769662.

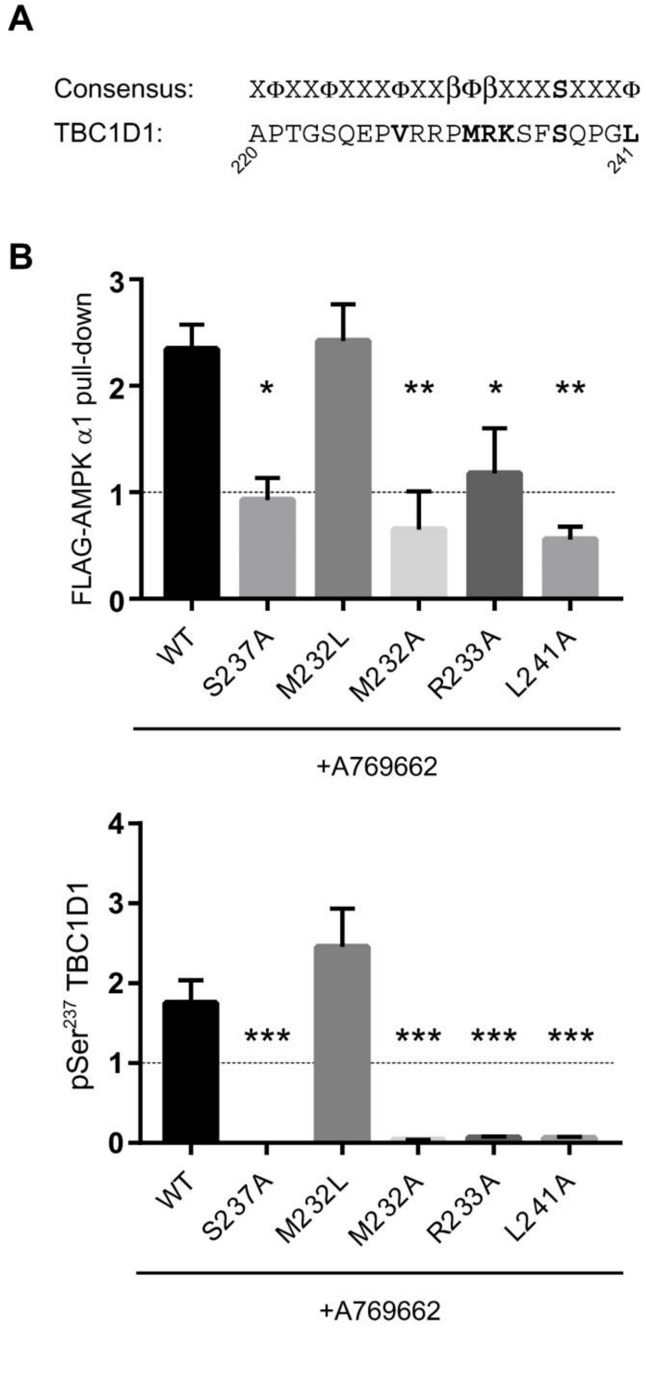

Figure S4. Kinase dead AMPK-α1 has enhanced binding to TBC1D1. (A) Expression of proteins as validation of CRISPR/Cas9 α1/α2 double knockout (DKO) compared to wild-type Flp-In HEK293 cells (WT). (B) Validation of AMPK activity in basal or A769662 (25 μM, 30 min) treated CRISPR/Cas9 α1/α2 double knockout (DKO) HEK293 cells stably expressing either WT or Kinase Dead (KD) FLAG-AMPK-α1. (C) Immobilised GST-His or GST-PTB1+2-His proteins were incubated with lysis buffer only (-) or lysates from DKO HEK293 cells stably expressing either WT or K D FLAG-AMPK-α1 and washed complexes analysed. (D) Quantitation of data in C, normalised to GST-PTB1+2 and expressed in terms of the WT condition. Mean ± SEM; 4 independent experiments; two-tailed t-test ****p<0.0001.

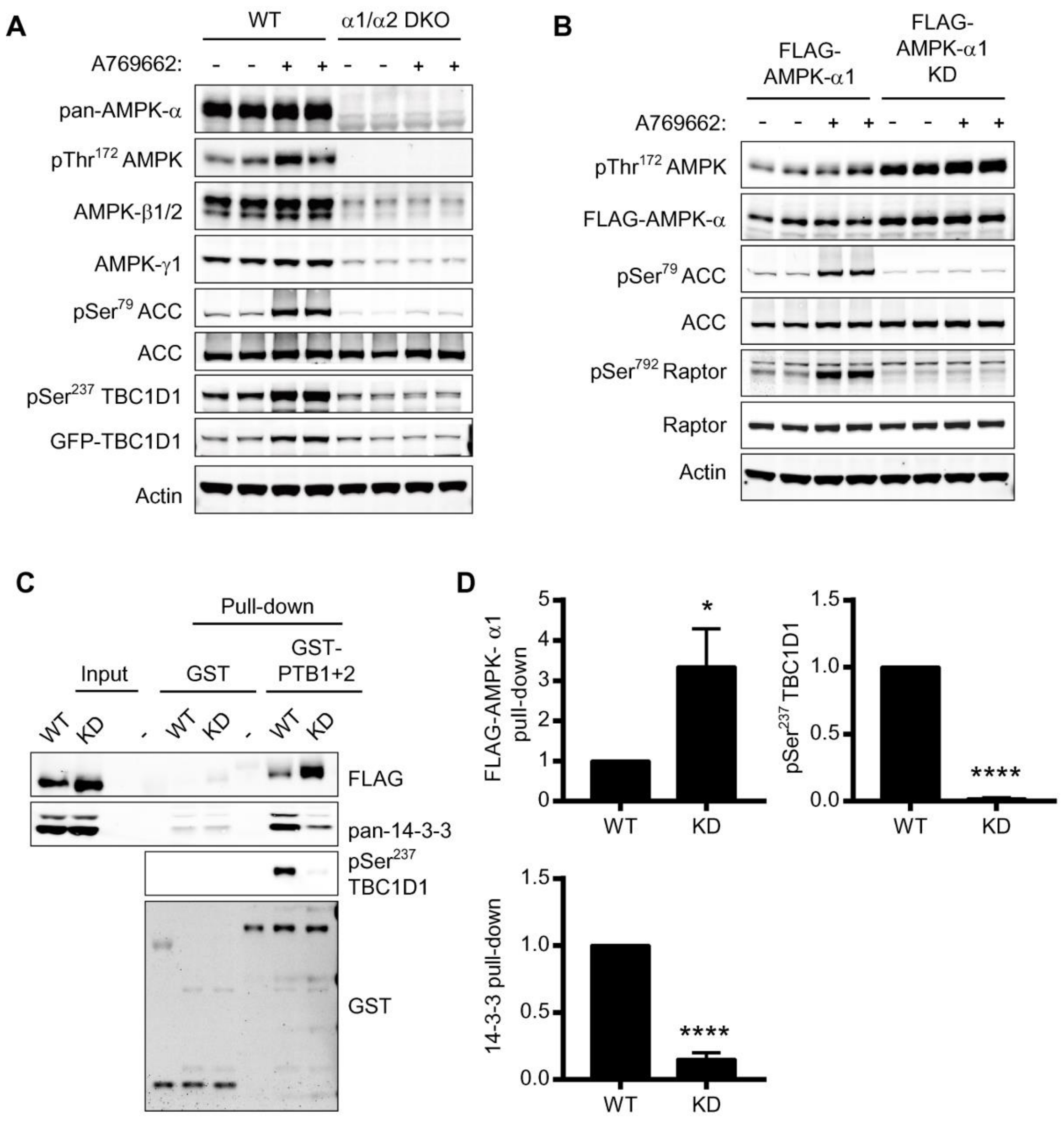

Figure S5. Ser^{237} phosphorylation of R125W mutant in FLAG-AMPK-α2 cells. (A) Quantitative fluorescence-based Western blot analysis of phosphorylation of TBC1D1, AMPK and ACC and total proteins from Flp-In HEK293 cells stably expressing FLAG-AMPK-α2 and transiently expressing either GFP-TBC1D1 or GFP-TBC1D1 R125W. Cells had been serum starved (4-5 h) prior to addition of STO-609 (25 µM, 30 min) and subsequent treatment with either vehicle or A769662 (25 µM) for the times indicated. (B) Quantitation of data shown in A in GFP-TBC1D1 (black circles) and GFP-TBC1D1 R125W (white diamonds) expressing cells; Ser^{237} phosphorylation normalized to GFP-tagged protein expression; AMPK phosphorylation normalized to FLAG-AMPK-α1 expression; ACC phosphorylation normalized to total ACC expression. All displayed as a fold over basal phosphorylation. Mean ± SEM; 4 independent experiments.

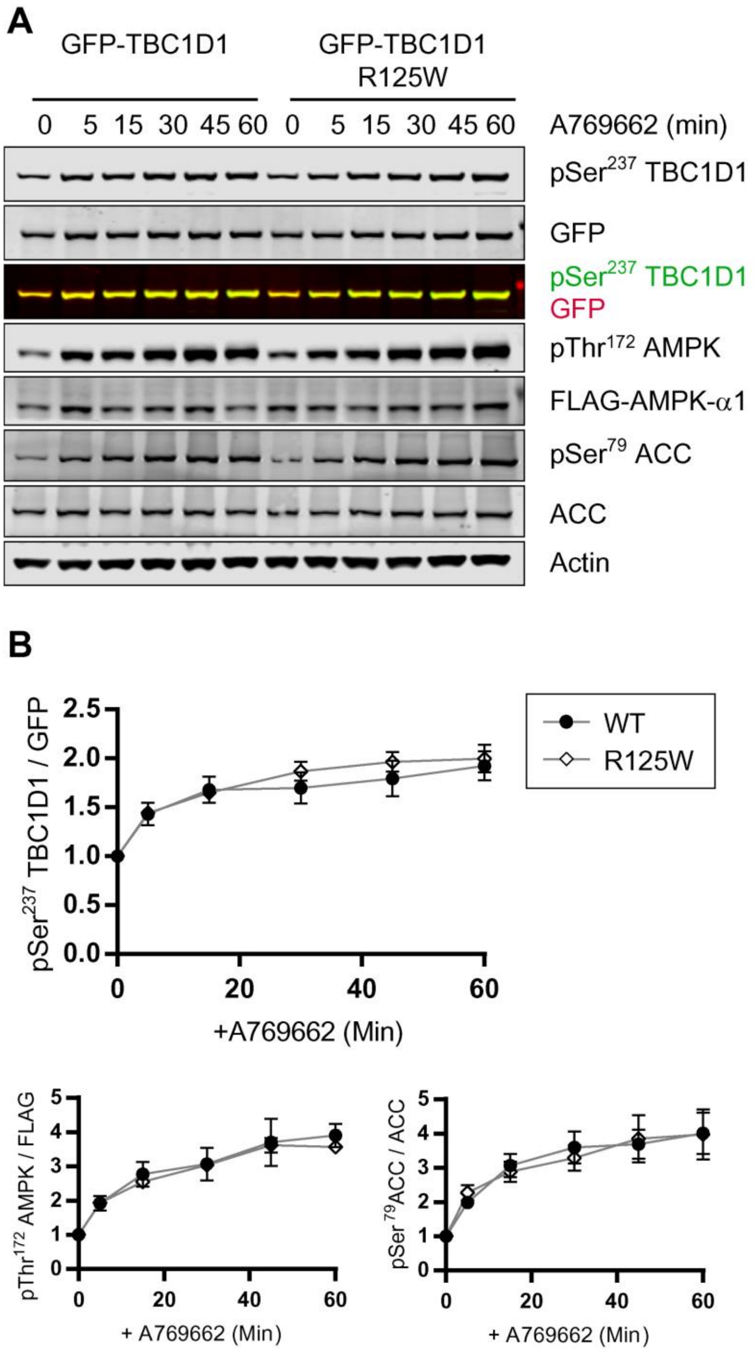

Supplement: Supplementary Figures [file BCJ-475-2969-s1.pdf]
